# Impact of a moulded pureed diet on taste, appearance, recognisability, and overall liking among patients in an acute hospital

**DOI:** 10.3389/fnut.2023.1248779

**Published:** 2023-09-19

**Authors:** Calvin Jun Yan Lam, Quan Quan Phua, Emily Yiting Guo, Isaac Kwee Mien Sia

**Affiliations:** Department of Rehabilitation, National University Hospital, Singapore, Singapore

**Keywords:** pureed diet, moulded diet, nutrition, taste, appearance, recognisability

## Abstract

**Introduction:**

Hospital meals potentially influence patients’ nutritional, physical, and emotional well-being during their admission. Patients on pureed diets report poorer meal satisfaction, due to taste, appearance, and recognisability, potentially impacting on their nutritional status. This study compared whether a moulded pureed diet made from modified maize starch led to improved taste, appearance, recognisability, and overall liking, compared to an unmoulded pureed diet made from potato starch in an acute hospital.

**Methods:**

Patients on texture-modified diets were recruited and presented with two pureed diets – unmoulded and moulded. Participants were asked to identify meat and vegetable dishes prior to eating. After the meal, participants indicated their diet preference in terms of appearance, taste, and overall liking.

**Results:**

145 participants were recruited, of which 126 completed data collection. 86% correctly identified moulded meat dishes, 69% correctly identified moulded vegetable dishes, with an overall 77% accuracy in identifying moulded puree side dishes. On unmoulded puree side dishes, participants correctly identified 25% of meat dishes, 4% of vegetable dishes, with an overall accuracy of 14%. In terms of preference, the moulded puree was preferred, with 81% for appearance, 76% for taste and 75% for overall preference. When participants had differing preferences for appearance and taste (e.g., prefers unmoulded puree appearance and moulded puree taste), 95% of them subsequently aligned their overall preference with their taste preference (i.e., overall preferred moulded pureed diet). This suggests that taste has a stronger influence on overall preference compared to appearance.

**Discussion:**

Findings indicate that a moulded pureed diet made from modified maize starch led to improved recognisability, taste, appearance, and overall liking compared to an unmoulded pureed diet made from potato starch. Taste had a stronger influence on overall preference compared to appearance. These findings capture patient preferences and may have implications on how hospital pureed diets may be improved, potentially improving patient nutrition and health outcomes.

## 1. Introduction

A hospital meal is an essential part of a patient’s care, and has the potential to influence a patient’s nutritional, physical, and emotional well-being (1). However, more than 50% of hospital patients do not eat a full meal (2, 3). Texture modified meals, which include pureed diets, are often prescribed during a patient’s hospital stay as part of their recovery. There are two main reasons patients are placed on pureed diets. Firstly, pureed foods are easier and safer to consume for patients with difficulties masticating and swallowing harder foods, due to ageing or related medical conditions (4, 5). Secondly, pureed diets may be offered to patients post-operations as a therapeutic diet in their recovery (6).

Pureed diets present great challenges with respect to nutrition and appeal (7). It is known that patients on pureed diets have poorer meal satisfaction than other patients on regular diets (7–9).

It has consistently been found that patients on pureed diets do not enjoy their food (10). Thickeners are frequently used to increase the viscosity of pureed foods to improve swallowing safety. However, it was found that patients could taste the thickeners in pureed diets, which diluted the flavour of pureed foods (7).It is unclear what types of thickeners have greater impact on the taste of pureed foods. Cassens et al. (11) used several different commercially prepared thickeners and everyday products (e.g., rice cereal, corn starch, gelatin) to thicken pureed food which was reported to improve taste. However, it was not reported whether certain types of thickeners were more effective than others in improving taste, and which ones they were. Other studies investigating the taste of moulded and unmoulded pureed foods focused on participants’ reports and ratings about taste, but provided limited information about the types of thickener used (10, 12, 13).

In this study, we will examine whether using tasteless modified maize starch leads to improved taste compared to pureed foods made with potato starch.

Pureed foods are generally considered unappealing due to their appearance (7, 14). In a study by Keller and Duizer (7), interviewed participants expressed how the appearance of their foods made them feel undignified and stigmatised compared to others who received “normal” foods. Of the studies that have examined the impact of the appearance of pureed foods on consumer satisfaction, findings have been inconclusive due to methodological differences and small sample sizes. Two studies investigating 20 acute hospital patients and 16 adults with swallowing impairment, respectively, found no significant differences in their ratings of food appearance on unmoulded and moulded pureed foods (10, 13). However, Lepore et al. (9) found that the appearance of unmoulded pureed foods was preferred over that of moulded pureed foods by young and old healthy adults.

In this study, we will examine whether moulded or unmoulded pureed foods are more visually appealing to patients.

Patients on pureed diets have reported that being unable to recognise their food is one of the reasons why they do not finish their food (10). Keller and Duizer (7) further reported that consumers of pureed foods wanted to be able to identify their food by taste if not appearance, and they preferred foods with distinctive tastes for ease of identification.

In this study, we will examine whether moulded pureed foods made with tasteless modified maize starch leads to improved recognisability compared to unmoulded pureed foods made with potato starch.

Studies have shown that taste and appearance share a complex relationship that influences eating behaviour (13, 15). While one study found that taste has more influence over patient preferences for pureed foods than appearance (7), another study found that a visual difference in pureed food was perceived as a taste difference (13). These findings reaffirm that taste and appearance are factors that interlink and may affect food preferences.

Taste, appearance, and recognisability are three major reasons why meal satisfaction is affected in patients on pureed diets. Existing pureed diets in the National University Hospital (NUH), where this research was conducted, are unmoulded and made using potato starch. The research diet aims to address the factors of taste, appearance, and recognisability by using moulded pureed foods made from tasteless modified maize starch. Improving taste, appearance, and recognisability will potentially improve nutritional, physical, and emotional well-being of patients on pureed diets.

This study investigates whether a moulded puree diet made from modified maize starch will lead to improved taste, appearance, recognisability, and overall liking compared to an unmoulded puree diet made from potato starch in an acute hospital.

It is hypothesised that:

- In terms of taste, moulded pureed diets made with tasteless modified maize starch will be preferred compared to unmoulded pureed diets made with potato starch.
- In terms of appearance, moulded pureed diets will be preferred compared to unmoulded pureed diets.
- In terms of recognisability, moulded pureed diets made with tasteless modified starch will be preferred compared to unmoulded pureed diets made with potato starch.
- In terms of overall liking, moulded pureed diets made with tasteless modified maize starch will be preferred compared to unmoulded pureed diets made with potato starch.

## 2. Materials and methods

### 2.1. Setting

The research setting was an acute hospital in Singapore, National University Hospital (NUH). The acute setting was chosen given the authors’ links to the site and therefore accessibility to potentially suitable participants for study recruitment. The authors were also interested to examine findings from an acute setting given that previous research on moulded and unmoulded pureed diets were mostly conducted in longer term care settings.

### 2.2. Data sampling

Purposive sampling was selected. Inclusion criteria for participants included: (a) at least 21 years of age, (b) speaks English or Mandarin, (c) on a modified diet up to point of survey administration, and (d) score of <8 (i.e., normal cognition) on 6-item Cognitive Impairment Test (6CIT). The 6CIT consists of 6 questions – 1 memory, 2 calculation, and 3 orientation. It has been shown by Katzman et al. (16) to discriminate among mild, moderate, and severe cognitive deficits. The tool has also been demonstrated to be valid in detecting milder forms of dementia, and is compared favourably to the Mini Mental State Examination due to its brevity and ease of use (17). Exclusion criteria for participants included: (a) do not speak English or Mandarin, (b) medically unstable as determined by the medical team, (c) known food refusal and/or poor oral intake, (d) history of head and neck cancer with chemoradiation therapy, (e) on therapeutic diet (i.e., meal plans that control the intake of certain foods or nutrients), (f) visual impairment, and (g) illiteracy combined with significant hearing impairment without remediation (i.e., hearing aids).

Participants were recruited during their inpatient acute hospital stay. Participants on modified diets (either as prescribed by a Speech-Language Therapist, or given based on participants or caregiver reports of premorbid diets at home) were identified on the hospital’s electronic meal ordering system. They were subsequently screened to ensure they met the above inclusionary and exclusionary criteria. For participants on follow-up with dieticians, their dieticians were consulted to ensure they were suited to participate in the study.

### 2.3. Data collection

Data was collected using questionnaires administered at participants’ bedsides. Prior to the research trial, participants completed the 6-item Cognitive Impairment Test (6CIT) to determine their level of cognition. Informed consent was taken at that point. If participants had impaired cognition (score of >8 on 6CIT), they were automatically excluded from their study. Participants were assigned a number for recording purposes, and confidentiality and anonymity were maintained during the course of the study. Recruited participants were then interviewed during their mealtimes within their admission (usually the following day). The questionnaire was administered in two parts – before meal and after meal. Before the meal, participants were presented with NUH’s unmoulded pureed diet and asked to identify the meat and vegetable sides. They were then presented with the moulded pureed diet and asked to do the same. They were also asked to indicate which diet they preferred in terms of appearance. Subsequently, patients were asked to consumed no more than half of each meal before indicating which diet they preferred on the variables of taste and overall liking. Participants were only required to select either the research diet or the current NUH diet for the variables of appearance, taste, and overall liking. Questionnaires were presented in either English or Mandarin.

**Figure 1:**
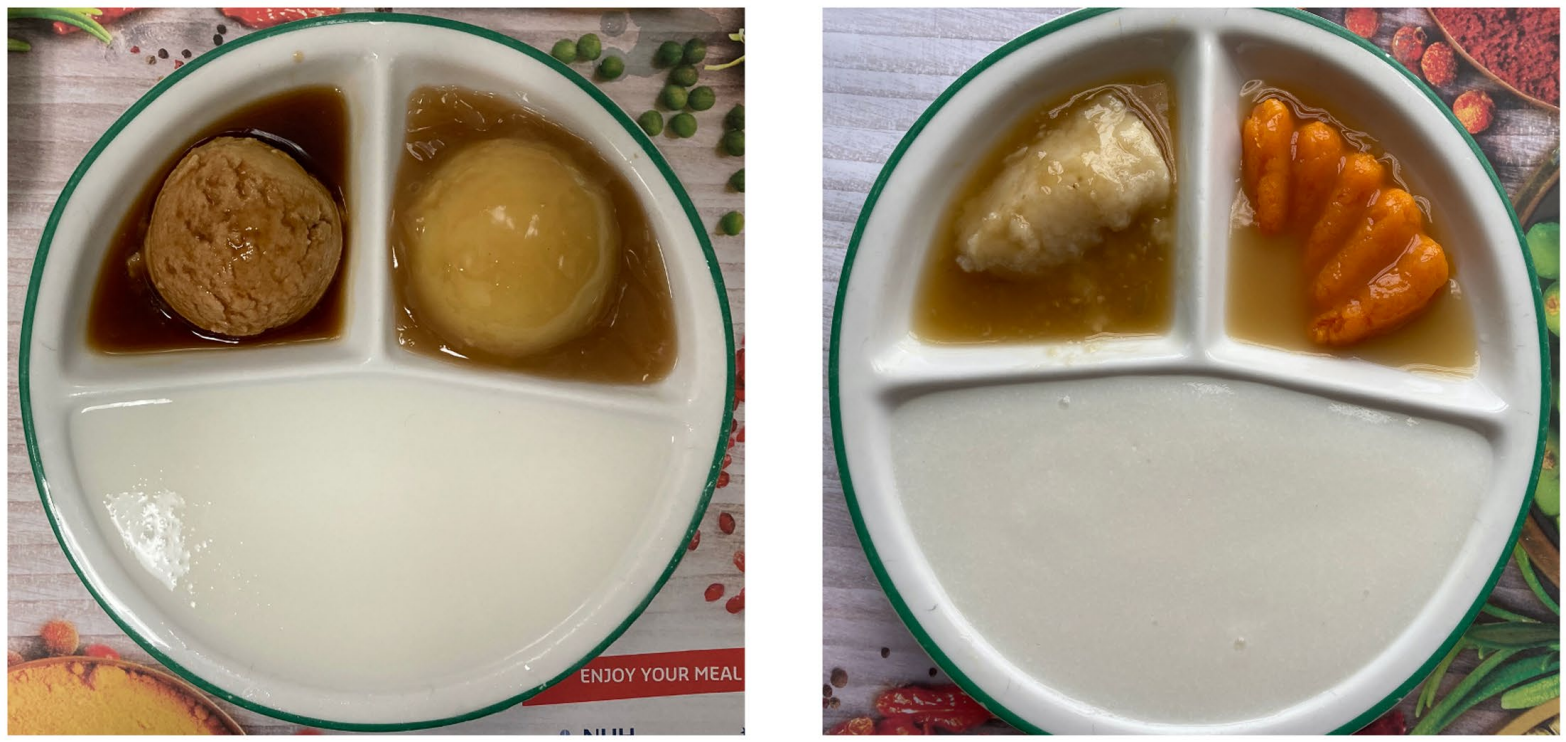
NUH pureed diet (left) and research diet (right).

Both diets were prepared by Sodexo Singapore within the NUH kitchen. The unmoulded pureed diet was the current NUH pureed diet, which consists of porridge, one meat and one vegetable dish. The meat and vegetables were blended with potato starch using an industrial blender to achieve a pureed consistency. They were then served in a rounded scoop on a plate, with a standard portion size of 200 g per dish. The moulded pureed diet was the research diet, which also consisted of porridge, one meat and one vegetable dish. Cooked meat and vegetables were blended with modified maize starch to achieve a pureed consistency and shaped using food moulds based on the dish (e.g., chicken moulded to the shape of a chicken drumstick). The modified maize starch used was Food Mold Thickener, designed specifically for use with pureed food moulding. Four moulds were used for the duration of the research – two vegetable moulds (carrot and broccoli), and two meat moulds (chicken and fish). The food moulds were subsequently frozen and stored in walk-in freezers with temperatures maintained at −18°C and below, and steamed before serving. The moulded foods were subsequently unmoulded onto plates, and steamed at 100°C. Porridge, identical to what is served in the current NUH pureed diet, was added to the plates. Both trays of food were delivered to the wards in food warmers at temperatures ranging from 75 to 85°C and subsequently served to participants. Ingredients chosen for the research were matched to the food moulds – carrot, broccoli, chicken, and fish. Both diets are shown in Figure 1 below.

The study was approved by the National Healthcare Group Domain Specific Review Board (NHG DSRB Ref: 2019/00564).

### 2.4. Data analysis

The primary outcome measure was overall liking of the unmoulded and moulded pureed diets. The secondary outcome measures were taste, appearance, and food recognisability. These variables have been shown to be important sensory properties that influence appetite and food intake (18, 19).

Descriptive statistics were used to detail the profile of participants recruited for the study. Chi-squared tests were also used to determine any significant associations between gender, age, ethnicity, medical discipline and the factors of appearance, taste, and overall liking. The data was also analysed using percentage calculations across four categories: percentages of participants who (a) correctly identified meat and vegetable dishes on the moulded and unmoulded puree diets, (b) preferred the appearance of the moulded versus unmoulded puree diets, (c) preferred the taste of the moulded versus unmoulded puree diets, and (d) preferred the moulded versus unmoulded pureed diets overall.

## 3. Results

Table 1 details the profiles of the participants recruited for the study. 145 participants were recruited, of which 126 completed data collection. The other 19 participants did not complete data collection as they either discharged or transferred to another institution prior to the research trial, or were medically unsuitable to proceed with the study at the point of research.

**Table 1:** Profile of participants across gender, age, race, and medical discipline.

	*n*	%
Gender		
Male	75	59.5
Female	51	40.5
Age (*m* = 67)		
30–59	32	25.4
60 and above	94	74.6
Race		
Chinese	97	77.0
Malay	16	12.7
Indian	6	4.8
Others	7	5.5
Patient distribution across disciplines		
Cardiac	17	13.5
ENT	3	2.4
General Medicine	23	18.3
General Surgery	26	20.6
Gastro	6	4.8
Oncology	13	10.3
Neurology	31	24.6
Others	7	5.5

A chi-square test of independence showed there was no significant association between gender and appearance preference, x^2^ (1, *n* = 126) = 0.0174, *p* = 0.895, between gender and taste preference, x^2^ (1, *n* = 126) = 2.12, *p* = 0.146, and between gender and overall liking, x^2^ (1, *n* = 126) = 1.61, *p* = 0.204.

A chi-square test of independence also showed no significant association between age range (60 and above versus 59 and below) and appearance preference, x^2^ (1, *n* = 126) = 0.326, *p* = 0.568, between age and taste preference, x^2^ (1, *n* = 126) = 0.172, *p* = 0.678, and between age and overall liking, x^2^ (1, *n* = 126) = 0.281, *p* = 0.596.

Finally, a chi-square test of independence also showed no significant association between medical discipline and appearance preference, x^2^ (7, *n* = 119) = 7.42, *p* = 0.283, between discipline and taste preference, x^2^ (7, *n* = 119) = 2.58, *p* = 0.859, and between discipline and overall liking, x^2^ (7, *n* = 119) = 1.63, *p* = 0.950.

The results demonstrated no significant association between appearance, taste, and overall preference and the factors of gender, age, or medical discipline.

Table 2 summarises the participants’ questionnaire results. 86% of participants correctly identified moulded meat dishes, 69% correctly identified moulded vegetable dishes, with an overall 77% accuracy in identifying moulded pureed side dishes. On unmoulded puree side dishes, participants correctly identified 25% of meat dishes, 4% of vegetable dishes, with an overall accuracy of 14%. In terms of preference, the moulded puree was preferred, with 81% for appearance, 76% for taste and 75% for overall preference. When participants had differing preferences for appearance and taste (e.g., preferred unmoulded puree appearance and moulded puree taste), 95% of them subsequently aligned their overall preference with their taste preference (i.e., overall preferred moulded puree diet).

**Table 2:** Questionnaire results: participant accuracy in terms of recognisability, preferences in terms of appearance, taste, and overall liking.

	A moulded pureed diet		B unmoulded pureed diet	
	*n*	%	*n*	%
Accurate recognition (meat)	108	85.7	32	25.4
Accurate recognition (veg)	87	69.0	5	4.0
Appearance preference	10	80.9	24	19.2
Taste preference	96	76.1	30	25.0
Overall liking preference	95	75.4	31	24.8

## 4. Discussion

Findings indicated that recognisability was improved on the moulded pureed diet compared to the unmoulded pureed diet. Given that moulding the pureed diet to its food shape improved recognisability, care institutions may wish to consider moulding purees to food shapes given that patients prefer foods they can recognise (7, 10).

This study also found that patients preferred the appearance of the moulded pureed diet made from tasteless modified maize starch compared to the unmoulded pureed diet made from potato starch. This contrasts with other studies where no differences in visual preferences were observed (10, 13), and Lepore et al.’s (9) study where the appearance of unmoulded foods was preferred. However, part of these inconsistencies could be attributed to the fact that the definition of “moulded” pureed was different across studies – with Stahlman et al. (13) and Lepore et al. (9) moulding their pureed foods into the shape of the food, while Farrer et al. (10) described their moulded food as three-dimensional.

Results from this study also indicated that the moulded pureed diet made from tasteless modified maize starch led to improved taste compared to an unmoulded pureed diet made from potato starch. As it is known that patients are able to taste the thickeners in their food which leads to reduced enjoyment of food taste (7), it is likely that the use of tasteless modified maize starch thickener in this study contributed to the improvement in taste perceived by participants. However, it is notable that a number of participants described the research diet as “sticky,” “chewy,” “gel-like” and generally less smoothly pureed compared to the unmoulded diet made from potato starch. These differences were perceived positively by a few participants, but generally more negatively by the majority of participants who made such observations. This could have been a result of the type of thickener used, or from the method of preparation by our facility’s food service department.

It is known that taste and appearance share a complex relationship that influences eating behaviour (13, 15). This was similarly observed in our research findings, with taste having a stronger influence over overall preference compared to appearance. When participants had differing preferences in diet type for taste and appearance domains, 95% of them subsequently aligned their overall preference with their taste preference. This is in alignment with the findings of Keller and Duizer (7) where taste was also found to have a stronger influence over food preferences compared to appearance. By contrast, unlike Stahlman et al. (13) who found that visual differences in food were perceived as taste differences, some participants in this study were disappointed by the taste of the moulded puree diet because they had expected better taste after observing the improved appearance of the moulded puree diet.

The findings therefore suggest that the factors of taste, appearance, and recognisability are important when considering pureed diets. Improving these could potentially help improve food intake and potentially reduce the risk of their nutritional status deteriorating in the course of their hospital stay. This could potentially help with reducing the future risk of malnutrition, with its prevalence estimated at 20 to 50% in hospitalised patients (20).

### 4.1. Limitations and future opportunities

The scope of this study did not allow for longer term data collection across multiple timepoints, partially due to the fact that the length of stay of patients in an acute setting is variable and often unpredictable. In this study, a single meal was administered with patients eating and comparing both the moulded and unmoulded puree diets at the same time in a single timepoint, with patient preference data collected. In a previous study by Farrer et al. (10), participants were surveyed on their preferences after a period of 2 weeks where participants had the moulded and unmoulded puree diets for 1 week each. Other studies (9–11, 21) also considered oral intake and weight gain as indicators of patient preferences and impact of moulded puree on overall health outcomes. As such, future studies may also consider collaboration with Dietitians as well as a longer-term methodology, where the impact of a moulded puree diet on oral intake and nutritional status may be studied, and patient preference findings may be more reflective of patient preferences over time.

While the research compared percentages of the participants’ preferences across the various factors, the study could have objectively compare which factor was the most important, and to examine the relation between the variables to observe for significant associations using a chi-square test. Given that the survey only included preference for which diet, rather than rating each diet on a scale, the preferred statistical comparison could not be achieved.

Our study used purees that were moulded to the shape of the food. Across the literature, it was observed that different studies defined “moulded purees” differently, with two studies (9, 13) found by the authors to also have used purees moulded to a specific food shape. Given that there were contradictory findings between this study and previous studies in terms of the impact of appearance on patient diet preferences, future investigation into whether moulding purees to their food shape will improve patient’s appetites is warranted.

### 4.2. Conclusion

The findings from this research show that a moulded puree diet made from tasteless modified maize starch led to improved recognisability, taste, appearance, and overall liking compared to an unmoulded puree diet made from potato starch, which supports the hypotheses made for the study. These findings capture patient preferences and may have implications on how hospital puree diets may be improved, potentially improving patient nutrition and health outcomes. In particular, the findings suggest that using a tasteless thickener can improve the taste and overall liking of a pureed diet.

## Data availability statement

The raw data supporting the conclusions of this article will be made available by the authors, without undue reservation.

## Ethics statement

The studies involving humans were approved by National Healthcare Group Domain Specific Review Board. The studies were conducted in accordance with the local legislation and institutional requirements. The participants provided their written informed consent to participate in this study. Written informed consent was obtained from the individual(s) for the publication of any potentially identifiable images or data included in this article.

## Author contributions

CL was responsible for the overall conduct of the study in NUH. CL and QP worked together in recruitment, data collection, data analyses, and writing up the results of the study. IS and EG provided supervision and guidance during the planning phase of the project – including study design and DSRB application. EG also provided assistance with the administrative aspects of the research (e.g., grant claims) and guidance in writing up the study report. All authors contributed to the article and approved the submitted version.

## Funding

The research was funded by the National University Hospital Services Centre Grant Seed Funding (NUHSCGSF/2019/10).

## Conflict of interest

The authors declare that the research was conducted in the absence of any commercial or financial relationships that could be construed as a potential conflict of interest.

## Publisher’s note

All claims expressed in this article are solely those of the authors and do not necessarily represent those of their affiliated organizations, or those of the publisher, the editors and the reviewers. Any product that may be evaluated in this article, or claim that may be made by its manufacturer, is not guaranteed or endorsed by the publisher.
